# An epithelial–mesenchymal transition-like synovial stromal-remodeling programme associated with knee osteoarthritis pain: Exploratory transcriptomic discovery and spatial analysis

**DOI:** 10.1371/journal.pone.0346948

**Published:** 2026-09-30

**Authors:** Diovanni de Paula Ferreira, Rafael de Negreiros Botan, Wagner R. Martins, Iruena M. Kessler

**Affiliations:** 1 Programa de Pós-Graduação em Ciências Médicas, Universidade de Brasília, Brasília, Brazil; 2 Department of Oncology, UnB, Brasília, Brazil; 3 Faculdade de Educação Física e Fisioterapia, UnB, Brasília, Brazil; 4 Department of Neurosurgery, UnB, Brasília, Brazil; Anhui University of Chinese Medicine, CHINA

## Abstract

**Background:**

Pain is the defining symptom of knee osteoarthritis (OA), yet its molecular correlates in synovial tissue remain incompletely understood.

**Methods:**

Across publicly available synovial transcriptomic datasets, we derived an exploratory pain-associated gene program by differential expression in synovium stratified by pain severity (GSE99662, n = 10; high vs low pain), characterized its pathway enrichment, tested specificity against an OA-versus-healthy contrast (GSE89408, n = 50), related it to inflammatory and fibrotic axes (GSE283079, n = 36 OA), and examined a larger, independent patient-level whole-transcriptome spatial cohort (Philpott et al. 2025 [1]; GSE248454; 32 knee-OA patients, 16 more- vs 16 less-pain; intimal-lining, sublining and perivascular compartments, one region per compartment per patient). The spatial analysis applied a limit-of-quantification detection filter and permutation of residuals (Freedman–Lane) from models adjusting for age and region cellularity, alongside correlation-aware competitive testing (CAMERA). This analysis plan was specified post hoc.

**Results:**

Discovery yielded an exploratory program of 38 genes (35 up-regulated). An epithelial–mesenchymal transition (EMT)-like stromal/remodeling signature — interpreted in synovium as matrix remodeling rather than literal epithelial transition — was the only Hallmark set of 50 significantly enriched (NES = 1.885, p_adj_ = 0.0022), although at n = 10 it did not survive exact phenotype-label permutation (p = 0.226). In the spatial cohort only 4,584 of 18,695 targets were above the detection limit in a typical region. After filtering, the discovery-derived Hallmark-EMT programme was positively associated with more pain in sublining regions (Holm-adjusted p = 0.010 and 0.005 at ≥5% and ≥10% detection across the discovery-derived hypotheses × compartments), persisting after adjustment for age and cellularity and against gene sets matched on abundance and detection, and carried by matrix, adhesion and TGF-β-associated transcripts. Three limits are reported with equal weight: the effect on the module scale was modest and imprecise (+0.30 z-units, 95% CI −0.06 to +0.65); an undirected scan of all 50 Hallmark sets across three compartments yielded no set surviving correction (EMT q = 0.105); and a within-patient contrast did not confirm that the association differs between compartments (p = 0.196). The 35-gene signature did not replicate in any compartment; a nominal unadjusted perivascular association did not survive adjustment, attenuated primarily by age. The programme was not enriched in OA-versus-healthy synovium.

**Conclusions:**

An EMT-like stromal-remodeling transcriptional programme is associated with worse patient-level knee pain in sublining synovium. The evidence is exploratory and method-dependent, the compartment difference is not established, and the specific discovery signature does not generalize.

## Introduction

Pain is the defining symptom of knee osteoarthritis (OA), yet its molecular basis in synovial tissue remains poorly understood. Clinical pain severity in OA correlates imperfectly with structural damage, and synovial inflammation and remodeling are increasingly recognized as pain-relevant features beyond cartilage-centric pathology [1]. A small pain-stratified transcriptomic study of OA synovium identified gene-expression changes associated with pain severity [2], and site-specific sampling within the knee has revealed differential fibroblast phenotypes across painful and non-painful synovial sites — a related but distinct construct from between-patient pain severity [3]. The synovial fibroblast compartment is functionally heterogeneous, comprising lining and sublining states that contribute differently to matrix remodeling, immune-cell recruitment and inflammatory signaling [4].

Here we derive an exploratory pain-associated synovial transcriptomic program, characterize its dominant pathway, test its specificity against generic OA pathology and its relationship to inflammatory and fibrotic axes, and examine whether it is recoverable in a larger, patient-level, whole-transcriptome spatial cohort. Because the discovery cohort is small (n = 10) and the spatial analysis is observational and post hoc, we treat the entire study as hypothesis-generating and report the specifications under which our conclusions do and do not hold.

## Methods

### Analysis-plan status

The discovery analyses were specified before the spatial cohort was examined. The spatial analysis plan reported here — detection filtering, covariate adjustment, permutation scheme and multiplicity strategy — was specified post hoc, after earlier analyses of the same cohort had been performed. It is not a pre-registered confirmatory analysis, and we report it as such throughout. Two hypotheses were carried forward from the discovery cohort: the Hallmark-EMT set (the only one of 50 with FDR < 0.05) and the 35-gene pain-UP signature. The synovial compartment was not pre-specified: the sublining had originally been selected on the basis of a CXCL12^+^ localization claim that we no longer maintain. We therefore test all three compartments and correct across the resulting family.

### Ethics

This study analysed only publicly available, de-identified datasets deposited in NCBI GEO. No human participants were recruited, no identifiable information was accessed, and no original experimental data were generated. Institutional review board approval and informed consent were therefore not required for this work; the primary studies obtained their own approvals as described in their respective publications.

### Dataset selection

Human knee-OA synovial transcriptomic datasets were identified in NCBI GEO and the literature (searched June 2026) using “knee osteoarthritis”, “synovium/synovial”, “pain”, and “transcriptome/RNA-seq/spatial”. Datasets were included if they profiled human knee synovial tissue or knee synovial cells and supported one of the analytical roles in Table 1; a patient-level pain measure was required for the discovery and spatial roles. A fixed seed (20260312) was used.

**Table 1:** Datasets used in this study.

Dataset	Tissue / model	Design	N	Platform	Pain measure	Role
GSE99662	Knee OA synovium	High vs low pain	10	bulk RNA-seq	VAS	Discovery (exploratory)
GSE248454	Knee OA synovium	More vs less pain; spatial, 3 compartments	32 patients / 96 ROIs	GeoMx WTA spatial	KOOS	Spatial cohort
GSE89408	Knee synovium	OA vs healthy	50	bulk RNA-seq	—	Disease dissociation
GSE283079	Knee OA synovium	OA subtypes	41 (36 OA analysed)	bulk RNA-seq	—	Inflammatory/fibrotic context
GSE176308	Knee OA synovium	scRNA-seq, 3 patients	4,086 cells	scRNA-seq	painful sites	Qualitative context only (not analysed)
GSE157364	Synovial fibroblasts + macrophages	ACP + /- TNF, 4 donors	32	bulk RNA-seq	—	Exploratory (Discussion only)
GSE176223	Knee OA synovium	Painful vs non-painful sites	12 patients / 23	bulk RNA-seq	site pain map	Portability (Supplement)

### Discovery analysis (GSE99662)

Counts were filtered (≥ 10 reads in ≥ 3 samples) and analyzed with DESeq2 and apeglm log_2_ fold-change shrinkage [5,6]. The exploratory program (38 genes: 35 up; 3 down) was defined at FDR < 0.10 and |log_2_FC| > 0.5. For preranked enrichment, genes were ranked by the apeglm shrunken log_2_ fold-change divided by its posterior SD; fgsea was run against MSigDB Hallmark, GO Biological Process, KEGG and Reactome [7] with Benjamini–Hochberg adjustment. Hallmark-EMT enrichment was re-evaluated under exact phenotype-label permutation across all 252 balanced 5-versus-5 relabelings.

### Spatial cohort (GSE248454)

Q3-normalized GeoMx Whole-Transcriptome Atlas counts and segment annotations were obtained from GEO (18,695 targets × 96 regions; 32 patients, 16 more- vs 16 less-pain; compartments: intimal lining, sublining and perivascular). The fluorescent channels (CD68, CD45, αSMA) guided placement of each region within the tissue architecture; the collected area contains the full local cell mixture and is not a sorted population. All patients were profiled on a single tissue microarray slide, so there is no batch structure. Each patient contributed exactly one region per compartment, and all tests were run within compartment (16 vs 16 patients).

**Detection filtering.** The deposited matrix is Q3-normalized whereas the per-region limit of quantification (LOQ) is on the raw scale. Raw counts were reconstituted as raw = Q3 / NormalizationFactor and this reconstruction was validated against the raw count matrix deposited in the probe-level QC export of the same series (maximum absolute deviation 3.6 × 10^−12^; 100% of values identical to within 10^−6^; Pearson r = 1.0000000). A target was called detected in a region when its reconstructed raw count reached that region’s LOQ. Analyses were repeated in two universes — targets detected in ≥ 5% and in ≥ 10% of regions — and both are reported; neither is designated primary.

**Covariate balance and adjustment.** Clinical and technical covariates were compared between pain groups. Age band (p = 0.001) and region nuclei count (perivascular p = 0.005; sublining p = 0.052) were imbalanced; sex, BMI, area, reads, sequencing saturation, Q30 metrics, normalization factor and LOQ were not (S1 Table). Because label permutation is not valid under imbalance, inference used Freedman–Lane permutation of residuals from a reduced model containing age (as a factor) and log_2_ nuclei count, with a single permutation applied across all genes so that gene–gene correlation is preserved (B = 10,000). Four adjustment models are reported — unadjusted, age only, cellularity only, and both — so that attenuation can be attributed rather than asserted. Because cellularity may be a consequence of the tissue phenotype rather than a technical confounder, adjustment for it is presented as a sensitivity analysis.

**Test statistic and multiplicity.** The test statistic was the mean rank of the gene set within the gene-wise t-statistic ranking for the pain contrast. Correlation-aware competitive testing (CAMERA, inter-gene correlation estimated from the data) is reported alongside for every specification. Multiplicity was handled with one stated strategy per layer: for the two discovery-derived hypotheses across three compartments (family of six), Holm correction, one-sided in the direction observed in discovery; for the exploratory scan of all 50 Hallmark sets across three compartments (family of 150), Benjamini–Hochberg, two-sided, since no direction was pre-specified.

**Composition.** Per-region cell-type proportions were estimated by deconvolution (SpatialDecon with the tumour-derived safeTME reference; 95/96 regions converged) and by a marker-based fibroblast identity score. Both are reported as sensitivity analyses only. We report differences in composition as estimates with confidence intervals rather than as significance tests, because a non-significant difference does not establish absence of confounding.

### Statistical analysis

Analyses used R 4.5.3 and Bioconductor 3.22 (limma, fgsea, msigdbr 26.1.0, SpatialDecon). Effect sizes: NES, Spearman ρ and partial r, Cliff’s δ, Hodges–Lehmann differences with 95% confidence intervals. Monte-Carlo intervals are reported for the one-sided permutation p-values. The exact Hallmark-EMT gene list and package versions are archived with the analysis code.

## Results

### An exploratory pain-associated program enriched for stromal remodeling

Differential expression of high- versus low-pain synovium (GSE99662) yielded 38 genes (35 up-regulated, 3 down-regulated) at FDR < 0.10 and |log_2_FC| > 0.5 (Fig 1; sensitivity to alternative thresholds in S2 Table). Under the apeglm shrunken-log_2_FC/SE ranking specified for the discovery analysis, the only significantly enriched Hallmark pathway was an EMT-like stromal/remodeling signature (NES = 1.885, p_adj_ = 0.0022; Fig 1C); in synovium this is interpreted as stromal extracellular-matrix remodeling rather than literal epithelial–mesenchymal transition [8]. The enriched landscape is ranking-statistic-dependent: under a DESeq2 Wald-statistic ranking, EMT remained significantly and positively enriched (NES = 1.59, p_adj_ = 0.0033) while additional Hallmarks also reached significance — notably inflammatory-response and interferon-γ terms enriched in the *negative* direction. We therefore do not claim a categorical absence of inflammatory enrichment, and note that inflammatory terms, where significant, were depleted rather than enriched with pain. The program is gene-list-cutoff-dependent: under stricter thresholds the list contracted to 24 genes at FDR < 0.05/|log_2_FC| > 0.5, 13 genes at FDR < 0.10/|log_2_FC| > 1, and 8 genes at FDR < 0.05/|log_2_FC| > 1. Importantly, at n = 10 the EMT enrichment did not reach significance under exact phenotype-label permutation across all 252 balanced relabelings (Welch-t p = 0.226; limma moderated-t p = 0.214), which is why inference rests on the independent cohort below. Quality control did not indicate gross technical failure (S1 Fig and S2 Fig).

**Fig 1:**
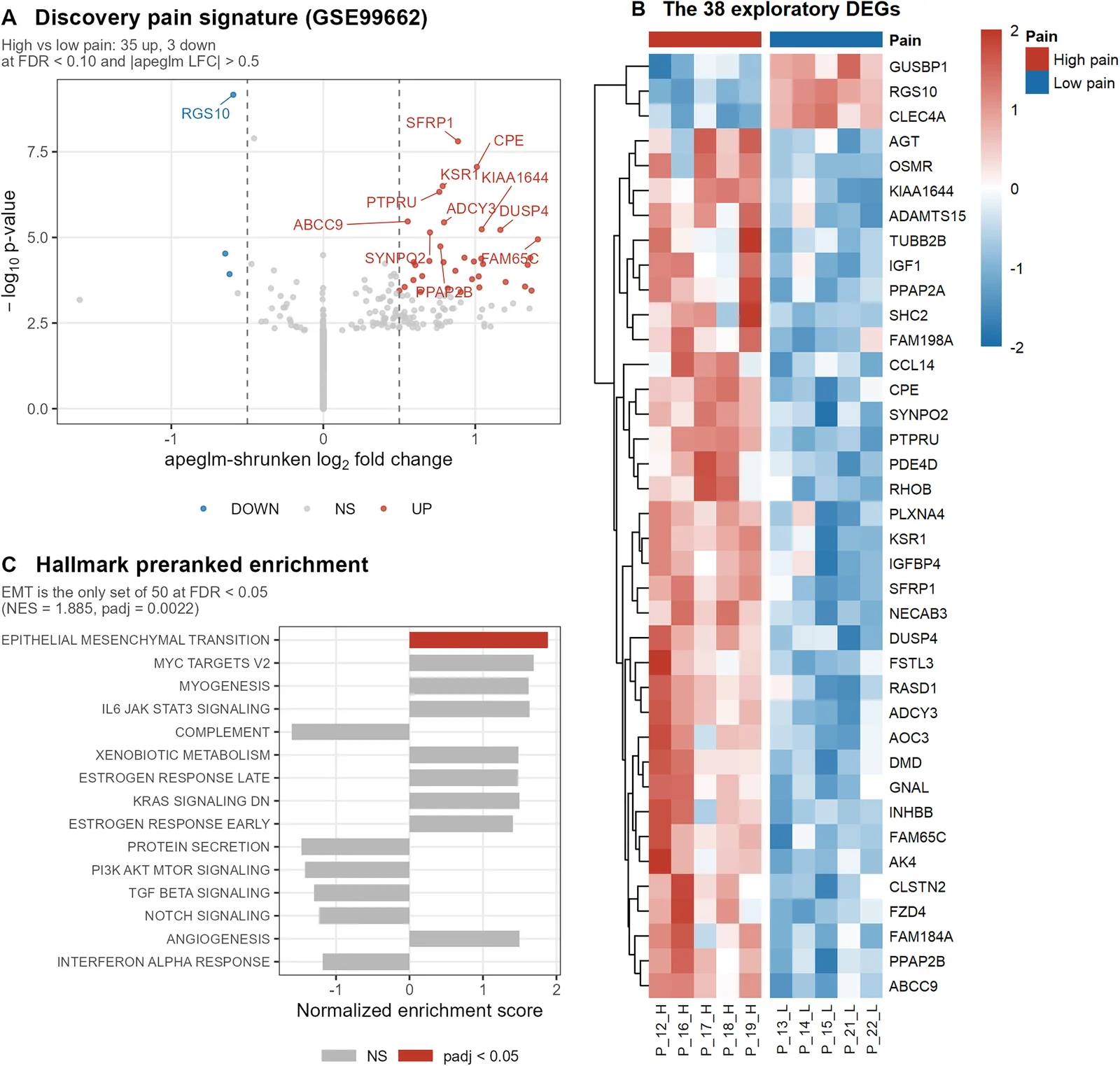
Discovery of an exploratory pain-associated synovial program (GSE99662). (A) Volcano plot (high vs low pain, n = 10). The x-axis shows apeglm-shrunken log_2_ fold-changes, the same scale on which the exploratory DEG set was defined (FDR < 0.10 and |apeglm-shrunken log_2_FC| > 0.5), so that the dashed guides at ±0.5 correspond to the coloured points. (B) Heatmap of all 38 DEGs, row-wise z-scores of variance-stabilised counts, samples ordered by pain group. (C) Hallmark preranked enrichment on the apeglm ranking; EMT is the only one of the 50 Hallmark sets reaching FDR < 0.05.

### Spatial cohort: Detection filtering changes the analytical substrate

A median of 4,584 of 18,695 targets (25%) were above the region-specific LOQ in a typical region. Filtering retained 14,600 targets (detected in ≥ 5% of regions) and 10,997 targets (≥ 10%). All spatial results below are reported in both universes, and we required agreement between them. Region placement across the tissue microarray, and the deconvolution-estimated cellular composition of each region, are shown in Fig 2A and Fig 2B.

**Fig 2:**
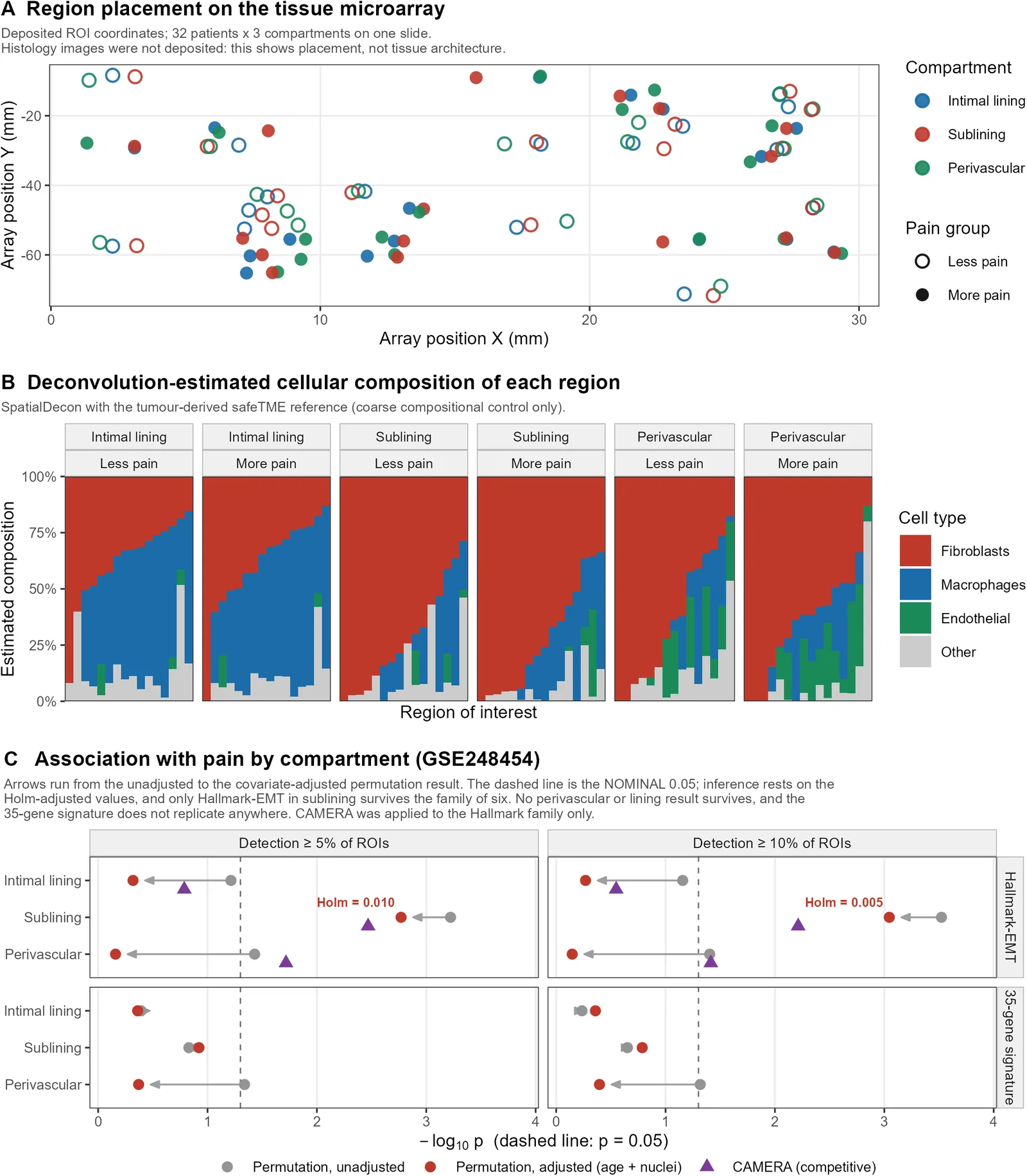
Spatial cohort (GSE248454). (A) Placement of the profiled regions across the tissue microarray, coloured by compartment and shaped by pain group. Histology images were not deposited with the dataset, so this panel shows region placement, not tissue architecture; the marker channels (CD68, CD45, αSMA) guided placement rather than sorting cells. (B) Deconvolution-estimated cellular composition of each region (SpatialDecon with the safeTME reference), which is a coarse compositional control rather than a cell-type assignment. (C) Association with pain by compartment for both discovery-derived hypotheses (Hallmark-EMT and the 35-gene pain-UP signature) in both detection universes. Grey points are the unadjusted permutation results and red points the same tests after Freedman–Lane adjustment for age and log_2_ nuclei count, with the arrow showing the shift; purple triangles are the corresponding CAMERA results, which were computed for the Hallmark family only. The dashed line marks the nominal p = 0.05 and is a reading aid, not the inferential threshold: inference rests on the Holm-adjusted values over the family of six, and the only two tests that survive them are labelled. Full numerical results, including Monte-Carlo intervals and the exploratory two-sided family-corrected q-values, are in Table 2.

**Table 2:** Spatial cohort (GSE248454): targeted and exploratory tests, all compartments and both detection thresholds. Four-model covariate decomposition, abundance/detection-matched null sets and leave-one-patient-out results are given in S3 Table. Targeted tests are the two discovery-derived hypotheses across three compartments (family of six, Holm, one-sided in the discovery direction). The exploratory scan is all 50 Hallmark sets across three compartments (family of 150, Benjamini–Hochberg, two-sided). Freedman–Lane permutation adjusts for age (factor) and log2 nuclei count; B = 10,000.

Detection	Hypothesis	Compartment	Unadj. p	Adj. p (one-sided)	MC 95%	Holm (family of 6)	Adj. p (two-sided)	CAMERA p	Exploratory q (150, two-sided)
≥5%	Hallmark-EMT	Intimal lining	0.0611	**0.4800**	[0.47007, 0.48975]	1.0000	0.9580	0.1632	0.993
≥5%	Hallmark-EMT	Sublining	0.0006	**0.0017**	[0.00091, 0.00260]	0.0102	0.0027	0.0034	0.102
≥5%	Hallmark-EMT	Perivascular	0.0371	**0.6938**	[0.68466, 0.70283]	1.0000	0.6071	0.0192	0.949
≥5%	35-gene signature	Intimal lining	0.4053	**0.4356**	[0.42575, 0.44529]	1.0000	0.8708	—	—
≥5%	35-gene signature	Sublining	0.1481	**0.1197**	[0.11330, 0.12612]	0.5984	0.2441	—	—
≥5%	35-gene signature	Perivascular	0.0459	**0.4267**	[0.41688, 0.43636]	1.0000	0.8563	—	—
≥10%	Hallmark-EMT	Intimal lining	0.0697	**0.5389**	[0.52907, 0.54871]	1.0000	0.9285	0.2829	0.989
≥10%	Hallmark-EMT	Sublining	0.0003	**0.0009**	[0.00035, 0.00158]	0.0054	0.0017	0.0061	0.105
≥10%	Hallmark-EMT	Perivascular	0.0395	**0.7136**	[0.70463, 0.72245]	1.0000	0.5718	0.0386	0.889
≥10%	35-gene signature	Intimal lining	0.5806	**0.4377**	[0.42785, 0.44739]	1.0000	0.8784	—	—
≥10%	35-gene signature	Sublining	0.2233	**0.1634**	[0.15610, 0.17069]	0.8169	0.3210	—	—
≥10%	35-gene signature	Perivascular	0.0482	**0.4020**	[0.39227, 0.41159]	1.0000	0.8097	—	—

### A sublining stromal-remodeling signal that is method-dependent

Of the six targeted tests (two discovery-derived hypotheses × three compartments), only Hallmark-EMT in the sublining was significant: p = 0.0017 (Holm 0.0102) at ≥5% detection and p = 0.0009 (Holm 0.0054) at ≥10%, with Monte-Carlo intervals of [0.00091, 0.00260] and [0.00035, 0.00158]. The remaining five tests had Holm-adjusted values of 0.60–1.00 (Table 2; Fig 2C).

The association is unlikely to be a simple consequence of transcript abundance, although we did not quantify how much of the displacement the residual mismatch below could account for: against 2,000 random gene sets matched on both mean abundance and detection rate and drawn from a pool excluding the EMT genes themselves, the observed set lay 8.6 SD (≥5%) and 8.9 SD (≥10%) above the matched null (p ≤ 0.0005). Matching was close but not exact, and the residual runs in favour of the observation rather than against it (mean log-abundance 3.61 in the set versus 3.41 in matched comparators), which we state rather than dismiss. No single patient dominated the statistic: under leave-one-patient-out, all 32 recomputed estimates remained above the permutation-null centre (9,829–10,610 versus a centre of 7,300 at ≥5%). We note that this establishes stability of the test statistic, not that significance is retained in every leave-one-out replicate, which we did not recompute. Adjustment did not act uniformly: for Hallmark-EMT in sublining, p was 0.0006 unadjusted, 0.0020 with age alone, 0.0001 with cellularity alone and 0.0017 with both, so neither covariate explains the signal.

Two caveats temper this. First, the adjusted effect on an interpretable scale is modest and imprecise: the difference in EMT module score between pain groups is + 0.30 z-units (95% CI −0.06 to +0.65) at ≥5% detection and +0.34 (−0.06 to +0.73) at ≥10%, with intervals obtained by bootstrapping patients. The competitive rank-based test is significant while the self-contained magnitude is not distinguishable from zero at 95% confidence; these address different questions and we report both. Second, one region has a recorded nuclei count of zero, and excluding it weakens the result from p = 0.0009 to p = 0.0063 (Holm 0.0375) — still significant, but more fragile than the headline value implies.

In the exploratory scan of all 50 Hallmark sets across the three compartments (150 tests, two-sided, Benjamini–Hochberg), no set survived correction in either universe; Hallmark-EMT in the sublining reached q = 0.105 (≥10%) and q = 0.102 (≥5%). Under the correlation-aware competitive test (CAMERA) the same contrast likewise did not survive family-wise correction (q = 0.41–0.49). The inference is therefore dependent on the null model and on the analytical family adopted, and the signal is hypothesis-generating rather than established. We note that within the targeted family the result does not depend on the use of a one-sided test: the two-sided adjusted p-values are 0.0027 (≥5%) and 0.0017 (≥10%), corresponding to Holm-adjusted values of 0.0162 and 0.0102. Every specification is given in Table 2 rather than summarised selectively.

### What drives the signal, and what the Hallmark label does and does not mean

Because “epithelial-mesenchymal transition” is a misleading label for a stromal tissue, we examined which genes carry the association. In the adjusted sublining analysis (>=10% detection), 123 of the 169 measurable Hallmark-EMT genes moved in the more-pain direction, and the strongest were matrix, adhesion and cytoskeletal transcripts: COL6A2 (t = 4.10), TPM2, VIM, COL5A1, TIMP3, HTRA1, ANPEP, ITGA5, PRRX1, ABI3 BP, DAB2, ITGB5, FN1, LGALS1, SPARC, FLNA, DPYSL3, FSTL1, LRP1, PCOLCE, SPOCK1, TNC, EFEMP2, BMP1 and LUM. Transforming-growth-factor-beta-associated members of the set (TGFB1, TGFBI, TGFBR3, BMP1, SERPINE1, THBS1, CCN2) were also predominantly positive.

Removing the 40 genes we annotated as structural extracellular-matrix components left the association essentially unchanged (129 genes; one-sided p = 0.0028, two-sided p = 0.0048 under the same patient-blocked residual permutation), indicating that the structural transcripts are not necessary for the result. We are careful about what this does and does not establish. It does not show independence from matrix biology, since several retained genes (FN1, SPARC, PCOLCE, TNC, SPOCK1, LUM, EFEMP2, TIMP3, HTRA1) are matrisome-associated. The categorisation is an author annotation applied post hoc and is provided as descriptive support only; a curated external matrisome annotation was not available in the gene-set package version used, which we regard as a limitation of this sub-analysis.

We also do not claim absence of an epithelial programme. The Hallmark-EMT set contains no epithelial markers by construction, so their absence here is a property of the set rather than an observation, and the small number of epithelial transcripts measurable elsewhere in the filtered matrix (KRT8, KRT18, CLDN4, CLDN7, MUC1) is too few to support a conclusion. What we can say is that no coordinated epithelial programme was demonstrated, and that the transcripts carrying the association are mesenchymal and matrix-related. Accordingly we describe the finding throughout as a stromal/mesenchymal remodeling programme captured by the Hallmark-EMT gene set, retaining the official set name for traceability while avoiding the literal epithelial reading. We do not attribute it to a specific cell type or state: the profiled regions contain fibroblasts, pericytes, smooth muscle, endothelium and macrophages, and neither cellular source nor a distinction between greater cell abundance and greater per-cell activation can be resolved with these data.

### The compartment difference itself is not established

Every patient contributed one region in each compartment, which allows a contrast formed entirely within a patient and therefore immune to age, sex, body-mass index and every other stable individual characteristic. Comparing each patient’s sublining score with the mean of that same patient’s lining and perivascular scores, the difference between pain groups was not significant (permutation p = 0.196 two-sided; Cliff’s delta = +0.28 in the expected direction). We therefore state the spatial claim conservatively: the association is detected in sublining regions, without confirmation that it differs between compartments.

### The 35-gene discovery signature does not replicate

After detection filtering and adjustment, the 35-gene pain-UP signature was not associated with pain in any compartment (Holm-adjusted 0.60–1.00). In particular, the nominal unadjusted perivascular association (p = 0.046) does not survive adjustment. The four-model decomposition shows that this attenuation is driven primarily by age rather than by cellularity: p moves from 0.046 unadjusted to 0.168 with age alone, but only to 0.063 with cellularity alone, reaching 0.427 with both. Because more-pain patients in this cohort are systematically younger (p = 0.001), the unadjusted perivascular association appears to have been substantially an age signal.

### Composition does not explain, and cannot exclude

Deconvolution-estimated sublining fibroblast proportion did not differ detectably between pain groups (0.778 vs 0.765), but the Hodges–Lehmann difference of +0.002 carries a 95% confidence interval of −0.173 to +0.175: the study cannot exclude compositional differences of ±17 percentage points, and we do not claim that composition has been controlled. The two adjustment schemes we examined disagree in the perivascular compartment (marker-based: direction reverses, p = 0.84; deconvolution-based: attenuated but not reversed, p = 0.078); both are reported.

### The program ranks low, but is not coherently reduced, in generic OA

In an OA-versus-healthy synovial contrast (GSE89408, n = 50), the pain-UP module was not positively enriched; the competitive test placed it significantly in the opposite direction (NES = −1.65, p_adj_ = 0.039; Fig 3A). A per-gene view (Fig 3B; per-gene values in S4 Table) shows that this is a relative ranking result rather than a coherent reduction of the programme: of the 30 programme genes measurable in this dataset, 16 are lower in OA and 14 are higher, the median difference is −0.04 log_2_ units (IQR −0.36 to +0.30), and only four genes reach FDR < 0.05 — in both directions (CLSTN2 −1.07 and INHBB −0.93 lower; OSMR +1.23 and PDE4D + 1.35 higher). The programme therefore ranks low relative to the remainder of the transcriptome without being coherently reduced.

**Fig 3:**
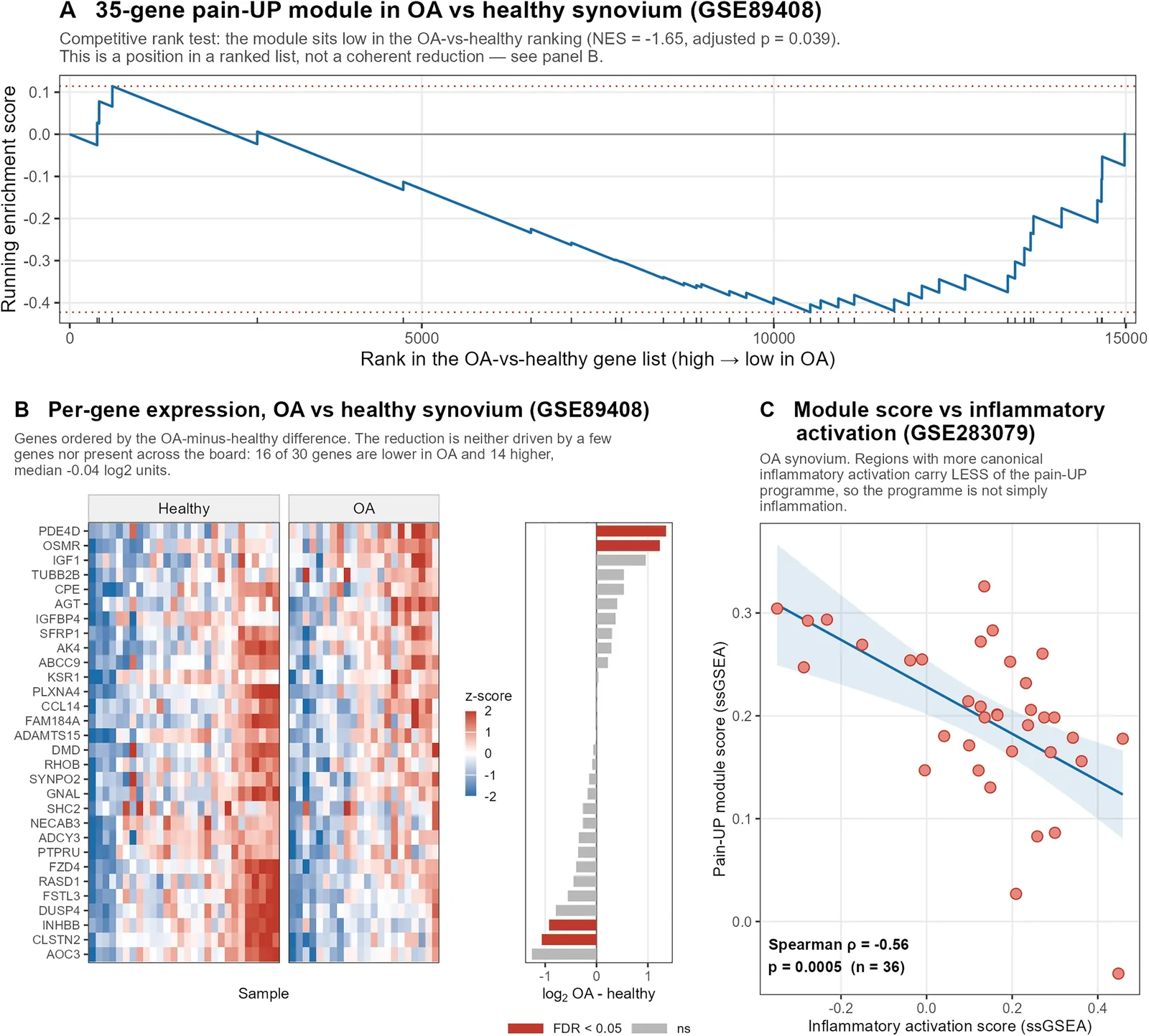
Specificity and context. (A) Competitive enrichment of the 35-gene pain-UP module in OA versus healthy synovium (GSE89408): the module ranks significantly low relative to the rest of the transcriptome (NES = −1.65, p_adj_ = 0.039). Panel B shows that this is a relative-ranking result and not a coherent reduction of the programme. (B) Per-gene heatmap of the programme in OA versus healthy synovium, with the per-gene OA-minus-healthy difference alongside, showing whether the reduction is driven by particular genes or is distributed across the set: 16 of 30 genes are lower and 14 higher in OA, median difference −0.04 log_2_ units, with four genes significant at FDR < 0.05 in both directions. (C) In OA synovium (GSE283079), the module score is inversely correlated with canonical inflammatory activation.

### Relationship to inflammatory and fibrotic axes

In an OA cohort (GSE283079, n = 36 OA), the 35-gene pain-UP module score was inversely correlated with canonical inflammatory activation (Spearman rho = −0.56, p = 5.3 x 10^−4^; Fig 3C). An association with the fibrotic/remodeling axis was not significant bivariately (rho = 0.19, p = 0.27) and emerged only after adjustment for inflammation (partial r = 0.34, p = 0.046), with a bootstrap confidence interval crossing zero; we therefore report it as non-robust.

## Discussion

We describe an exploratory transcriptomic program associated with pain severity in knee-OA synovium whose dominant signal is an EMT-like stromal/remodeling signature, and we examine whether it is recoverable in a larger, independent, patient-level spatial cohort. The honest summary is mixed: a positive sublining association is present and survives several demanding robustness checks, but it does not survive an undirected correction across the whole Hallmark collection, and the specific 35-gene signature does not replicate at all.

**Relation to the source study.** Philpott et al. [9] report that worse pain is associated with fewer immune-regulatory macrophages, macrophage exhaustion markers and reduced phagocytic capacity, established through histopathology, proteomics and single-cell RNA-seq. Our analysis of their spatial data reports higher ranking of several activation-associated programs, including TNF-α signaling, in more-pain regions. These observations interrogate different levels and can coexist: theirs is a cell-type-specific functional claim, ours a competitive ranking over whole-transcriptome regional profiles, in which loss of regulatory macrophages and higher ranking of inflammatory transcripts in the residual tissue are not mutually exclusive. We do not claim to have demonstrated that mechanism. Their study also reports microvascular dysfunction and perivascular oedema associated with worse pain, expanded fibroblast subsets and enrichment of neurovascular remodeling pathways, which are concordant with a stromal-remodeling reading of the same tissue. Analytical differences that may also contribute include normalization (we used the deposited Q3 matrix), detection filtering (applied here), the resulting gene universe, and the contrast (region-level more- versus less-pain within compartment).

**Limitations.** First, the discovery cohort is small (n = 10) and its EMT enrichment does not survive exact phenotype-label permutation, so it is hypothesis-generating. Second, the spatial analysis plan was specified post hoc; the compartment was not pre-specified, and although the gene set and its direction were fixed by the discovery cohort, the decision to test them in this particular way was made after earlier analyses of the same data. Third, and most importantly, the conclusion is specification-dependent: it holds under families restricted to discovery-derived hypotheses and under residual permutation, and fails under an undirected 50-set scan and under CAMERA. Fourth, two covariates were imbalanced between pain groups (age, region cellularity); the age adjustment has limited positivity, since one age band contains five more-pain and no less-pain patients, so the adjusted contrast extrapolates in that stratum. Fifth, cellularity may be a consequence of the phenotype rather than a confounder, in which case adjusting for it removes part of the signal of interest. Sixth, composition controls are approximate: safeTME is a tumour-derived reference and the background is approximated by the region LOQ. Seventh, no histology images were deposited with the spatial dataset, so the spatial context we can display is region placement and estimated composition, not tissue architecture. Finally, a within-knee site-level contrast (GSE176223) did not replicate the signature (S5 Table), consistent with site-level pain being a distinct construct from between-patient pain severity.

In summary, spatial data are compatible with a stromal/mesenchymal remodeling programme associated with worse patient-level knee pain, detected in sublining regions, but the evidence is method-dependent, the compartment difference is not established, and the specific discovery signature does not generalize. The findings are associative and motivate prospective, patient-level studies with pre-specified analysis plans.

### Declaration of generative AI use

During preparation the authors used AI tools to assist with code generation and statistical review; the authors reviewed and edited all content and take full responsibility for it.

## Supporting information

S1 Fig Discovery quality control – p-value distribution (GSE99662). Approximately uniform with a near-zero peak, consistent with a valid analysis.
(TIF)

S2 Fig Discovery quality control – principal-component analysis (GSE99662). PC1 = 44% and PC2 = 17% of variance; high- and low-pain groups do not separate.
(TIF)

S1 Table Covariate balance between pain groups (GSE248454). Patient-level clinical variables, slide and batch structure, and region-level technical metrics by compartment.
(DOCX)

S2 Table Threshold sensitivity of the discovery program (GSE99662). Differentially expressed genes (up/down) at FDR < 0.10 and |log2FC| > 0.5, FDR < 0.05 and |log2FC| > 0.5, FDR < 0.10 and |log2FC| > 1, and FDR < 0.05 and |log2FC| > 1.
(DOCX)

S3 Table Spatial cohort supporting analyses (GSE248454). Four-model covariate decomposition (unadjusted, age only, cellularity only, both) for both hypotheses in all three compartments and both detection thresholds; abundance- and detection-matched random gene-set results with matching diagnostics; leave-one-patient-out estimates; the within-patient compartment contrast; the leading-edge gene list with per-gene t statistics; and the sensitivity excluding the zero-nuclei region.
(DOCX)

S4 Table Per-gene expression of the pain-UP programme in OA versus healthy synovium (GSE89408). Log2 difference, p and FDR for each of the 30 measurable programme genes.
(DOCX)

S5 Table Site-level portability check (GSE176223). Paired within-patient comparison of painful versus non-painful sites; no gene set reaches FDR < 0.05.
(DOCX)
