# Supplementary material for: An epithelial–mesenchymal transition-like synovial stromal-remodeling programme associated with knee osteoarthritis pain: Exploratory transcriptomic discovery and spatial analysis
Source: PLoS One. 2026 Sep 30;21(9):e0346948. doi: 10.1371/journal.pone.0346948 (PMC13626332; doi:10.1371/journal.pone.0346948)
Supplement: Supplementary file 1 [file pone.0346948.s003.docx]

**S1 Table. Covariate balance between pain groups (clinical and technical), GSE248454. Patient-level clinical variables, slide/batch structure, and region-level technical metrics by compartment.**

| **Level** | **Variable** | **More pain** | **Less pain** | **p** | **Test** |
| --- | --- | --- | --- | --- | --- |
| Patient | Age band | 1.94 (SD 0.77) | 2.81 (SD 0.40) | 0.001 | Wilcoxon rank-sum |
| Patient | BMI band | 1.00 (SD 0.73) | 1.06 (SD 0.85) | 0.825 | Wilcoxon rank-sum |
| Patient | Sex (n female / male) | 10 / 6 | 9 / 7 | 1.000 | Fisher exact |
| Slide | Slide / scan batch | HP4_WTA (16) | HP4_WTA (16) | - | single slide, no batch |
| ROI - Intimal lining | Nuclei count | 106.0 (median) | 119.5 (median) | 0.266 | Wilcoxon rank-sum |
| ROI - Intimal lining | AOI surface area | 21636.9 (median) | 21664.2 (median) | 0.692 | Wilcoxon rank-sum |
| ROI - Sublining | Nuclei count | 89.5 (median) | 143.5 (median) | 0.052 | Wilcoxon rank-sum |
| ROI - Sublining | AOI surface area | 57497.2 (median) | 53632.8 (median) | 0.925 | Wilcoxon rank-sum |
| ROI - Perivascular | Nuclei count | 79.0 (median) | 184.5 (median) | 0.005 | Wilcoxon rank-sum |
| ROI - Perivascular | AOI surface area | 24931.1 (median) | 24375.6 (median) | 0.865 | Wilcoxon rank-sum |
