# Supplementary material for: An epithelial–mesenchymal transition-like synovial stromal-remodeling programme associated with knee osteoarthritis pain: Exploratory transcriptomic discovery and spatial analysis
Source: PLoS One. 2026 Sep 30;21(9):e0346948. doi: 10.1371/journal.pone.0346948 (PMC13626332; doi:10.1371/journal.pone.0346948)
Supplement: Supplementary file 2 [file pone.0346948.s004.docx]

**S2 Table. Threshold sensitivity of the discovery program (GSE99662). Differentially expressed genes at four combinations of FDR and apeglm-shrunken fold-change threshold.**

| **Criterion** | **Up** | **Down** | **Total** |
| --- | --- | --- | --- |
| FDR < 0.10 and \|apeglm log2FC\| > 0.5 | 35 | 3 | 38 |
| FDR < 0.05 and \|apeglm log2FC\| > 0.5 | 22 | 2 | 24 |
| FDR < 0.10 and \|apeglm log2FC\| > 1.0 | 13 | 0 | 13 |
| FDR < 0.05 and \|apeglm log2FC\| > 1.0 | 8 | 0 | 8 |
