# Supplementary material for: An epithelial–mesenchymal transition-like synovial stromal-remodeling programme associated with knee osteoarthritis pain: Exploratory transcriptomic discovery and spatial analysis
Source: PLoS One. 2026 Sep 30;21(9):e0346948. doi: 10.1371/journal.pone.0346948 (PMC13626332; doi:10.1371/journal.pone.0346948)
Supplement: Supplementary file 3 [file pone.0346948.s005.docx]

**S3 Table. Spatial cohort supporting analyses (GSE248454).**

**A. Four-model covariate decomposition (Freedman-Lane permutation, B = 10,000)**

| **Detection threshold** | **Hypothesis** | **Compartment** | **Model** | **One-sided p** | **Two-sided p** | **MC 95% low** | **MC 95% high** | **B** |
| --- | --- | --- | --- | --- | --- | --- | --- | --- |
| >= 10% of ROIs | 35-gene signature | Intimal lining | Unadjusted | 0.581 | 0.843 | 0.571 | 0.59 | 10000 |
| >= 10% of ROIs | 35-gene signature | Intimal lining | Age only | 0.308 | 0.618 | 0.299 | 0.317 | 10000 |
| >= 10% of ROIs | 35-gene signature | Intimal lining | Cellularity only | 0.658 | 0.687 | 0.649 | 0.668 | 10000 |
| >= 10% of ROIs | 35-gene signature | Intimal lining | Age + cellularity | 0.438 | 0.878 | 0.428 | 0.447 | 10000 |
| >= 10% of ROIs | 35-gene signature | Perivascular | Unadjusted | 0.0482 | 0.0926 | 0.044 | 0.0525 | 10000 |
| >= 10% of ROIs | 35-gene signature | Perivascular | Age only | 0.143 | 0.285 | 0.136 | 0.15 | 10000 |
| >= 10% of ROIs | 35-gene signature | Perivascular | Cellularity only | 0.064 | 0.131 | 0.0592 | 0.0689 | 10000 |
| >= 10% of ROIs | 35-gene signature | Perivascular | Age + cellularity | 0.402 | 0.81 | 0.392 | 0.412 | 10000 |
| >= 10% of ROIs | 35-gene signature | Sublining | Unadjusted | 0.223 | 0.444 | 0.215 | 0.231 | 10000 |
| >= 10% of ROIs | 35-gene signature | Sublining | Age only | 0.158 | 0.322 | 0.151 | 0.165 | 10000 |
| >= 10% of ROIs | 35-gene signature | Sublining | Cellularity only | 0.21 | 0.425 | 0.202 | 0.219 | 10000 |
| >= 10% of ROIs | 35-gene signature | Sublining | Age + cellularity | 0.163 | 0.321 | 0.156 | 0.171 | 10000 |
| >= 10% of ROIs | Hallmark-EMT | Intimal lining | Unadjusted | 0.0697 | 0.148 | 0.0647 | 0.0748 | 10000 |
| >= 10% of ROIs | Hallmark-EMT | Intimal lining | Age only | 0.364 | 0.725 | 0.354 | 0.373 | 10000 |
| >= 10% of ROIs | Hallmark-EMT | Intimal lining | Cellularity only | 0.109 | 0.216 | 0.103 | 0.116 | 10000 |
| >= 10% of ROIs | Hallmark-EMT | Intimal lining | Age + cellularity | 0.539 | 0.929 | 0.529 | 0.549 | 10000 |
| >= 10% of ROIs | Hallmark-EMT | Perivascular | Unadjusted | 0.0395 | 0.0819 | 0.0357 | 0.0434 | 10000 |
| >= 10% of ROIs | Hallmark-EMT | Perivascular | Age only | 0.189 | 0.389 | 0.182 | 0.197 | 10000 |
| >= 10% of ROIs | Hallmark-EMT | Perivascular | Cellularity only | 0.241 | 0.487 | 0.233 | 0.25 | 10000 |
| >= 10% of ROIs | Hallmark-EMT | Perivascular | Age + cellularity | 0.714 | 0.572 | 0.705 | 0.722 | 10000 |
| >= 10% of ROIs | Hallmark-EMT | Sublining | Unadjusted | 3e-04 | 7e-04 | 2.42e-05 | 0.000722 | 10000 |
| >= 10% of ROIs | Hallmark-EMT | Sublining | Age only | 7e-04 | 0.0019 | 0.00022 | 0.00131 | 10000 |
| >= 10% of ROIs | Hallmark-EMT | Sublining | Cellularity only | 3e-04 | 5e-04 | 2.42e-05 | 0.000722 | 10000 |
| >= 10% of ROIs | Hallmark-EMT | Sublining | Age + cellularity | 9e-04 | 0.0017 | 0.000345 | 0.00158 | 10000 |
| >= 5% of ROIs | 35-gene signature | Intimal lining | Unadjusted | 0.405 | 0.805 | 0.396 | 0.415 | 10000 |
| >= 5% of ROIs | 35-gene signature | Intimal lining | Age only | 0.35 | 0.69 | 0.34 | 0.359 | 10000 |
| >= 5% of ROIs | 35-gene signature | Intimal lining | Cellularity only | 0.457 | 0.908 | 0.447 | 0.466 | 10000 |
| >= 5% of ROIs | 35-gene signature | Intimal lining | Age + cellularity | 0.436 | 0.871 | 0.426 | 0.445 | 10000 |
| >= 5% of ROIs | 35-gene signature | Perivascular | Unadjusted | 0.0459 | 0.0894 | 0.0418 | 0.0501 | 10000 |
| >= 5% of ROIs | 35-gene signature | Perivascular | Age only | 0.168 | 0.339 | 0.161 | 0.175 | 10000 |
| >= 5% of ROIs | 35-gene signature | Perivascular | Cellularity only | 0.0628 | 0.121 | 0.058 | 0.0676 | 10000 |
| >= 5% of ROIs | 35-gene signature | Perivascular | Age + cellularity | 0.427 | 0.856 | 0.417 | 0.436 | 10000 |
| >= 5% of ROIs | 35-gene signature | Sublining | Unadjusted | 0.148 | 0.284 | 0.141 | 0.155 | 10000 |
| >= 5% of ROIs | 35-gene signature | Sublining | Age only | 0.115 | 0.232 | 0.109 | 0.122 | 10000 |
| >= 5% of ROIs | 35-gene signature | Sublining | Cellularity only | 0.149 | 0.293 | 0.142 | 0.156 | 10000 |
| >= 5% of ROIs | 35-gene signature | Sublining | Age + cellularity | 0.12 | 0.244 | 0.113 | 0.126 | 10000 |
| >= 5% of ROIs | Hallmark-EMT | Intimal lining | Unadjusted | 0.0611 | 0.128 | 0.0564 | 0.0659 | 10000 |
| >= 5% of ROIs | Hallmark-EMT | Intimal lining | Age only | 0.359 | 0.712 | 0.349 | 0.368 | 10000 |
| >= 5% of ROIs | Hallmark-EMT | Intimal lining | Cellularity only | 0.0852 | 0.164 | 0.0797 | 0.0907 | 10000 |
| >= 5% of ROIs | Hallmark-EMT | Intimal lining | Age + cellularity | 0.48 | 0.958 | 0.47 | 0.49 | 10000 |
| >= 5% of ROIs | Hallmark-EMT | Perivascular | Unadjusted | 0.0371 | 0.0782 | 0.0334 | 0.0409 | 10000 |
| >= 5% of ROIs | Hallmark-EMT | Perivascular | Age only | 0.177 | 0.356 | 0.17 | 0.185 | 10000 |
| >= 5% of ROIs | Hallmark-EMT | Perivascular | Cellularity only | 0.234 | 0.459 | 0.225 | 0.242 | 10000 |
| >= 5% of ROIs | Hallmark-EMT | Perivascular | Age + cellularity | 0.694 | 0.607 | 0.685 | 0.703 | 10000 |
| >= 5% of ROIs | Hallmark-EMT | Sublining | Unadjusted | 6e-04 | 8e-04 | 0.000162 | 0.00117 | 10000 |
| >= 5% of ROIs | Hallmark-EMT | Sublining | Age only | 0.002 | 0.0051 | 0.00114 | 0.00297 | 10000 |
| >= 5% of ROIs | Hallmark-EMT | Sublining | Cellularity only | 1e-04 | 1e-04 | 0 | 0.000369 | 10000 |
| >= 5% of ROIs | Hallmark-EMT | Sublining | Age + cellularity | 0.0017 | 0.0027 | 0.000915 | 0.0026 | 10000 |

**B. Abundance- and detection-matched random gene sets, EMT genes excluded from the pool**

| **Detection threshold** | **Compartment** | **EMT mean rank** | **Matched null (SD)** | **z** | **p** | **Abundance EMT vs null** | **Detection EMT vs null** |
| --- | --- | --- | --- | --- | --- | --- | --- |
| >= 5% of ROIs | Sublining | 10417 | 7675 (319) | +8.6 | 0.0005 | 3.485 vs 3.237 | 0.564 vs 0.549 |
| >= 5% of ROIs | Perivascular | 6654 | 7106 (299) | -1.5 | 0.9335 | 3.485 vs 3.236 | 0.564 vs 0.549 |
| >= 10% of ROIs | Sublining | 8013 | 5836 (244) | +8.9 | 0.0005 | 3.610 vs 3.408 | 0.608 vs 0.603 |
| >= 10% of ROIs | Perivascular | 4973 | 5308 (232) | -1.4 | 0.9265 | 3.610 vs 3.409 | 0.608 vs 0.603 |

**C. Leave-one-patient-out (adjusted model)**

| **Detection threshold** | **Compartment** | **Full-data mean rank** | **Null centre** | **Leave-one-out range** | **All 32 above null centre** |
| --- | --- | --- | --- | --- | --- |
| >= 5% of ROIs | Sublining | 10417 | 7300 | [9829, 10610] | TRUE |
| >= 5% of ROIs | Perivascular | 6654 | 7300 | [6102, 7357] | FALSE |
| >= 10% of ROIs | Sublining | 8013 | 5499 | [7585, 8173] | TRUE |
| >= 10% of ROIs | Perivascular | 4973 | 5499 | [4574, 5625] | FALSE |

**D. Within-patient compartment contrast: sublining minus the same patient's other compartments (Hallmark-EMT, >= 10% universe)**

| **Detection threshold** | **Hypothesis** | **Difference (more minus less)** | **Welch t p** | **Wilcoxon p** | **Hodges-Lehmann [95% CI]** | **Cliff delta** | **Permutation one-sided p** | **Permutation two-sided p** | **B** |
| --- | --- | --- | --- | --- | --- | --- | --- | --- | --- |
| >= 5% of ROIs | Hallmark-EMT | +0.109 | 0.1944 | 0.1835 | +0.097 [-0.063, 0.236] | +0.281 | 0.09870 | 0.19609 | 20000 |
| >= 5% of ROIs | 35-gene signature | -0.068 | 0.5765 | 0.6689 | -0.070 [-0.323, 0.198] | -0.094 | 0.71406 | 0.56732 | 20000 |
| >= 10% of ROIs | Hallmark-EMT | +0.107 | 0.2211 | 0.2099 | +0.088 [-0.077, 0.233] | +0.266 | 0.11014 | 0.21939 | 20000 |
| >= 10% of ROIs | 35-gene signature | -0.087 | 0.4843 | 0.5641 | -0.091 [-0.387, 0.182] | -0.125 | 0.75921 | 0.47793 | 20000 |

**E. Leading-edge genes, adjusted model, sublining (top 25 by t statistic)**

| **Rank** | **Gene** | **t statistic (adjusted model, sublining)** |
| --- | --- | --- |
| 1 | COL6A2 | 4.10 |
| 2 | TPM2 | 3.52 |
| 3 | VIM | 3.23 |
| 4 | COL5A1 | 3.08 |
| 5 | TIMP3 | 2.93 |
| 6 | HTRA1 | 2.80 |
| 7 | ANPEP | 2.76 |
| 8 | ITGA5 | 2.74 |
| 9 | PRRX1 | 2.71 |
| 10 | ABI3BP | 2.71 |
| 11 | DAB2 | 2.65 |
| 12 | ITGB5 | 2.63 |
| 13 | FN1 | 2.62 |
| 14 | LGALS1 | 2.62 |
| 15 | SPARC | 2.60 |
| 16 | FLNA | 2.59 |
| 17 | DPYSL3 | 2.58 |
| 18 | FSTL1 | 2.56 |
| 19 | LRP1 | 2.50 |
| 20 | PCOLCE | 2.49 |
| 21 | SPOCK1 | 2.49 |
| 22 | TNC | 2.38 |
| 23 | EFEMP2 | 2.33 |
| 24 | BMP1 | 2.30 |
| 25 | LUM | 2.27 |
| 26 | KRT8 | -1.53 |
| 27 | KRT18 | -1.89 |
| 28 | CLDN4 | 1.80 |
| 29 | CLDN7 | -0.17 |
| 30 | MUC1 | 0.18 |

**F. Sensitivity: excluding the single region with zero nuclei**

| **Compartment** | **ROIs retained** | **One-sided p** | **Empirical two-sided p** |
| --- | --- | --- | --- |
| Sublining | 31 | 0.00625 | 0.01415 |
| Perivascular | 32 | 0.71126 | 0.57837 |
| Intimal lining | 32 | 0.52667 | 0.94600 |
