# Supplementary material for: An epithelial–mesenchymal transition-like synovial stromal-remodeling programme associated with knee osteoarthritis pain: Exploratory transcriptomic discovery and spatial analysis
Source: PLoS One. 2026 Sep 30;21(9):e0346948. doi: 10.1371/journal.pone.0346948 (PMC13626332; doi:10.1371/journal.pone.0346948)
Supplement: Supplementary file 4 [file pone.0346948.s006.docx]

**S4 Table. Per-gene expression of the pain-UP programme in OA versus healthy synovium (GSE89408). Log2 difference, p and FDR for each of the 30 measurable programme genes.**

| **Gene** | **log2 difference (OA - healthy)** | **p** | **FDR** |
| --- | --- | --- | --- |
| AOC3 | -1.26 | 0.02 | 0.1 |
| CLSTN2 | -1.069 | 0.00517 | 0.0388 |
| INHBB | -0.925 | 0.00382 | 0.0382 |
| DUSP4 | -0.793 | 0.0665 | 0.222 |
| FSTL3 | -0.56 | 0.0361 | 0.155 |
| RASD1 | -0.452 | 0.254 | 0.523 |
| FZD4 | -0.395 | 0.337 | 0.574 |
| PTPRU | -0.362 | 0.227 | 0.523 |
| ADCY3 | -0.345 | 0.262 | 0.523 |
| NECAB3 | -0.263 | 0.23 | 0.523 |
| SHC2 | -0.262 | 0.45 | 0.695 |
| GNAL | -0.175 | 0.508 | 0.726 |
| SYNPO2 | -0.148 | 0.677 | 0.883 |
| RHOB | -0.087 | 0.72 | 0.9 |
| DMD | -0.067 | 0.902 | 0.993 |
| ADAMTS15 | -0.014 | 0.969 | 0.993 |
| FAM184A | 0.002 | 0.993 | 0.993 |
| CCL14 | 0.019 | 0.96 | 0.993 |
| PLXNA4 | 0.02 | 0.967 | 0.993 |
| KSR1 | 0.04 | 0.89 | 0.993 |
| ABCC9 | 0.221 | 0.643 | 0.877 |
| AK4 | 0.287 | 0.344 | 0.574 |
| SFRP1 | 0.301 | 0.463 | 0.695 |
| IGFBP4 | 0.372 | 0.322 | 0.574 |
| AGT | 0.402 | 0.253 | 0.523 |
| CPE | 0.529 | 0.217 | 0.523 |
| TUBB2B | 0.535 | 0.0556 | 0.208 |
| IGF1 | 0.963 | 0.0114 | 0.0685 |
| OSMR | 1.232 | 0.00359 | 0.0382 |
| PDE4D | 1.353 | 0.000987 | 0.0296 |
