# Supplementary material for: An epithelial–mesenchymal transition-like synovial stromal-remodeling programme associated with knee osteoarthritis pain: Exploratory transcriptomic discovery and spatial analysis
Source: PLoS One. 2026 Sep 30;21(9):e0346948. doi: 10.1371/journal.pone.0346948 (PMC13626332; doi:10.1371/journal.pone.0346948)
Supplement: Supplementary file 5 [file pone.0346948.s007.docx]

**S5 Table. Site-level portability check (GSE176223). Paired within-patient comparison of painful versus non-painful sites; no gene set reaches FDR < 0.05.**

| **Comparison** | **Gene set** | **Set size** | **NES** | **p** | **FDR** |
| --- | --- | --- | --- | --- | --- |
| All sites (paired) | pain_ALL | 25 | -1.194 | 0.204 | 0.204 |
| All sites (paired) | pain_UP | 24 | -1.231 | 0.172 | 0.204 |
| Early OA | pain_ALL | 26 | -1.319 | 0.106 | 0.106 |
| Early OA | pain_UP | 25 | -1.363 | 0.0798 | 0.106 |
| End-stage OA | pain_ALL | 25 | -1.12 | 0.295 | 0.295 |
| End-stage OA | pain_UP | 24 | -1.139 | 0.276 | 0.295 |
